# Overcoming Resistance to Therapies Targeting the MAPK Pathway in BRAF-Mutated Tumours

**DOI:** 10.1155/2020/1079827

**Published:** 2020-01-03

**Authors:** Emily L. Paton, Jacqueline A. Turner, Isabel R. Schlaepfer

**Affiliations:** Division of Medical Oncology, The University of Colorado School of Medicine, Anschutz Medical Campus, Aurora, CO, USA

## Abstract

Overactivation of the mitogen-activated protein kinase (MAPK) pathway is an important driver of many human cancers. First line, FDA-approved therapies targeting MAPK signalling, which include BRAF and MEK inhibitors, have variable success across cancers, and a significant number of patients quickly develop resistance. In recent years, a number of preclinical studies have reported alternative methods of overcoming resistance, which include promoting apoptosis, modulating autophagy, and targeting mitochondrial metabolism. This review summarizes mechanisms of resistance to approved MAPK-targeted therapies in BRAF-mutated cancers and discusses novel preclinical approaches to overcoming resistance.

## 1. Introduction

The mitogen-activated protein kinase (MAPK) cascade plays a critical role in cell survival, proliferation, and differentiation [1]. The components of the MAPK pathway are a highly conserved and ubiquitously expressed family of enzymatic kinases that phosphorylate many different target substrates [2, 3]. MAPK components are part of the large tiered phosphorylation cascade that includes RAS, RAF, MEK, and ERK kinases [4–6]. This tiered organization provides flexibility and adaptability, allowing a broad range of higher-order kinases to respond to the environment and control cellular function [7, 8]. Through transduction of signals from extracellular stimuli to downstream effector proteins within the cell, the MAPK pathway plays a major role in nearly every cellular process [1, 9].

**Figure 1:**
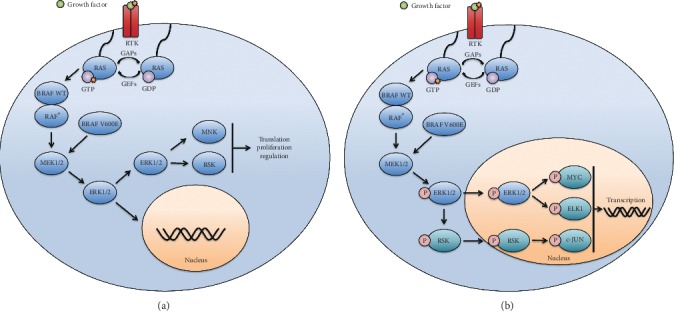
Mitogen-activated protein kinase (MAPK) pathway regulates nuclear and cytoplasmic activities. (a) Membrane receptor stimulation activates RAS GTPase which phosphorylates and activates RAF ⟶ MEK ⟶ ERK. BRAF forms homo- or heterodimers with other RAF-family proteins (ARAF or CRAF)^*∗*^ leading to MEK activation. BRAF^V600E^ is constitutively active and phosphorylates MEK independent of RAS activation and dimerization. ERK-specific phosphorylation regulates its localization. Cytoplasmic ERK regulates RSK and MNK to modulate cellular function including transcription, proliferation, and invasion. (b) Phosphorylated ERK may phosphorylate RSK, which can translocate to the nucleus. In the nucleus, other transcription factors are recruited to promote expression of growth and prosurvival proteins.

In healthy tissue, activation of the MAPK pathway arises from a variety of intracellular and extracellular stimuli, including metabolic stress, DNA damage, cytokines, and growth factors [9]. Typically, the MAPK pathway is stimulated by growth factors binding to receptor tyrosine kinases (RTKs). RTKs including epidermal growth factor receptor (EGFR), fibroblast growth factor receptor (FGFR), platelet-derived growth factor receptor (PDGFR), and vascular endothelial growth factor receptor (VEGFR) converge downstream onto MAPK [10–13]. Notably, hormone stimulation may also activate the MAPK pathway through the progesterone receptor (PgR) and estrogen receptor (ER) [14–16]. Progestin-bound PgR promotes rapid ER alpha/Src association to activate MAP [16]. Hormone-triggered MAPK signalling events have been well summarized by Giovannelli et al. [17]. In addition, stress-activated MAP kinases modulate the activity of several nuclear receptors, including androgen receptor (AR), estrogen receptor (ER), glucocorticoid receptor (GR), peroxisome proliferator-activated receptor (PPAR), and retinoic acid receptor (RAR) [18]. Overall, MAPK signalling is important for growth, development, and cell turnover across many tissue types. Canonical MAPK signalling results from membrane receptor stimulation that activates the small GTPase, RAS, leading to a kinase cascade that ultimately phosphorylates extracellular signal-related kinases (ERK) (Figure 1(a)) [19–22]. ERK has widespread cellular effects, activating target proteins in both the cytoplasm, including RSK and MNK (Figure 1(a)), and the nucleus, including c-JUN, MYC, and ELK1 (Figure 1(b)) [23–27]. ERK-MAPK signalling promotes cell survival, proliferation, and motility [28–31]. Notably, crosstalk between MAPK components and other pathways can enhance the effects of MAPK signalling and increase cell proliferation and oncogenic transformation [18].

Constitutive activation of the MAPK pathway, through overstimulation of receptors, RAS activation, or uncontrolled kinase activity, drives many human cancers [32]. Overactivation of BRAF, a RAF-family protein kinase and component of MAPK, is one of the most common events resulting in aberrant MAPK signalling [33]. BRAF is frequently mutated from GAG to GTG resulting in a valine to glutamic acid transition at amino acid position 600 in the activation loop of the BRAF kinase domain (BRAF^V600E^) [33–35]. This mutation forms a salt bridge between glutamic acid 600 and lysine 507 to promote an active, closed kinase conformation and facilitate catalysis [36]. In addition, the BRAF^V600E^ mutation destabilizes the hydrophobic interactions between the aspartic acid-phenylalanine-glycine (DFG) motif and the P-loop to promote the DFG motif to adopt an active inconformation resulting in autoactivation of the monomeric form of the BRAF kinase [37, 38]. The BRAF^V600E^ mutation constitutively activates the MAPK pathway independent of RAS stimulation and is the most common activating BRAF mutation [39–41]. However, other point mutations, gene fusions, splice site variants, and gene amplifications also lead to constitutive BRAF activity [42–45]. Mutations in BRAF are seen across many cancers including, but not limited to thyroid, melanoma, colon, squamous cell and hairy cell leukemia, and CNS-related malignancies [33, 46, 47]. Consequently, inhibiting BRAF kinase signalling is an attractive target, which can benefit patients across different cancer types (Table 1).

Vemurafenib, a kinase inhibitor, was first used in a phase 1 clinical trial for BRAF^V600E^-mutated metastatic melanoma in 2010 and was approved for use in 2011 [48, 49]. Despite improvements in overall survival, the majority of patients treated with BRAF inhibitors develop resistance and disease progression after 6-7 months [50, 51]. In 2018, the FDA approved the BRAF/MEK combination encorafenib plus binimetinib for BRAF^V600E^- and BRAF^V600K^-mutated metastatic melanoma based on more durable results from a Phase 3 clinical trial [52]. Combining BRAF and MEK inhibitors has improved the average progression-free survival of metastatic melanoma from 5.8 to 9.4 months but many patients still suffer from resistance to combination therapy [50]. Despite successes in melanoma, vemurafenib has variable efficacy in other BRAF^V600E^-mutated cancers such as thyroid, colorectal, and glial, with only a small fraction of patients responding [53–55]. While somewhat controversial, histological subtype, microsatellite instability, and other genetic alterations are proposed to contribute to variable responses to vemurafenib across cancers [55, 56].

Resistance to BRAF inhibition may be intrinsic or acquired, which is respectively observed as either no response to initial therapy or a response and later resistance to therapy. In this review, we will focus on the acquired resistance mechanisms precipitated by BRAF inhibition and alternative therapies that may overcome acquired resistance. Acquired resistance is a cellular alteration in addition to the BRAF^V600E^ mutation that facilitates tumour cells to grow despite BRAF inhibitor (BRAFi) therapy. Reactivation of MAPK signalling is the most common mechanism of acquired resistance to BRAFi therapy across cancer types [57]. However, recent studies have identified that overexpression of antiapoptotic genes, stimulation of autophagy, adenosine monophosphate protein kinase (AMPK) activation, alterations in the tumour microenvironment, and changes in metabolic flux also promote resistance and treatment failure [58–65]. Each section of this review describes the normal role, the aberrant activity resulting in acquired resistance, and potential approaches to targeting and overcoming these resistance mechanisms. Here, we review cellular adaptations that promote resistance to anti-MAPK therapy and summarize novel preclinical approaches to improving long-term patient responses across cancer types. We summarize recent promising preclinical therapies in this review; however, it is important to note that this is not a comprehensive list (Table 1).

## 2. Targeting Apoptosis in Cancer

### 2.1. Antiapoptotic BCL-2 Family Proteins Promote Resistance

**Figure 2:**
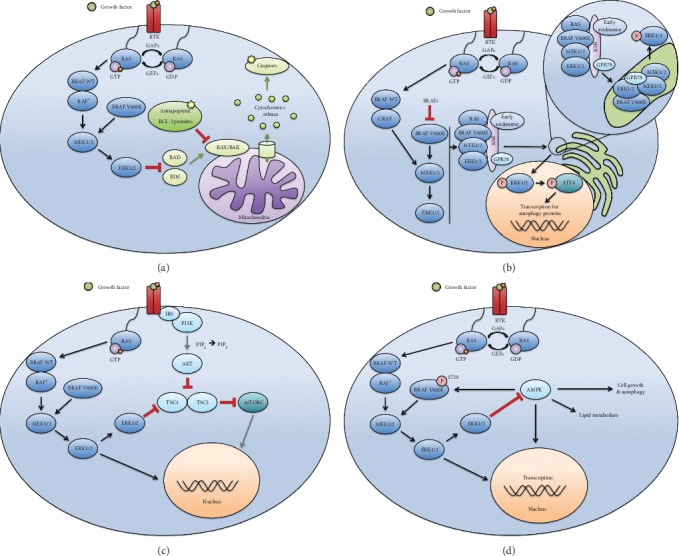
Noncanonical functions of MAPK are emerging targets for BRAF^V600E^-mutated cancers. (a) MAPK mediates apoptosis by inhibiting BH3-only proteins that activate proapoptotic BAX and BAK proteins. BCL-2 proteins inhibit caspase-mediated apoptosis by sequestering BAX and BAK to prevent release of mitochondrial cytochrome c. (b) MAPK proteins associate with KSR2, GRP78, and endosomes to localize to the ER. Translocation to the ER is required for ERK reactivation, ATF4 phosphorylation, and subsequent autophagy. (c) MAPK negatively regulates PI3K/AKT/mTOR through TSC1 dimers. The Insulin receptor substrate (IRS) recruits PI3K to the membrane resulting in activation and conversion of phosphatidylinositol-4,5-bisphosphate (PIP_2_) to phosphatidylinositol-3,4, 5-triphosphate (PIP_3_). The second messenger PIP_3_ promotes the activation of AKT. AKT inhibits the dimerization and activation of TSC1 and TSC2, leading to mTOR activation. (d) Downstream ERK inhibits the metabolic sensor AMPK, which modulates the MAPK pathway via phosphorylation of serine 729 on BRAF, likely representing a regulatory feedback loop. AMPK promotes catabolism, including lipid breakdown and autophagy.

Apoptosis, or programmed cell death, is a tumour suppression mechanism that is critical to healthy tissue homeostasis [66]. This program is a downstream effector pathway of the MAPK signalling cascade (Figure 2(a)) [67]. Apoptotic pathways are driven by the caspase protease family, which are proteolytically activated by either the intrinsic or extrinsic apoptotic pathway [68]. The intrinsic pathway (the “stress” or “mitochondrial” pathway) is mediated by pro- and antiapoptotic members of the B-cell lymphoma-2 (BCL-2) protein family and is implicated in tumourigenesis [69, 70]. Proapoptotic BCL-2 Associated X (BAX) and BCL-2 Antagonist Killer (BAK) form homo- and heterodimers stimulate the release of mitochondrial cytochrome c [71–73]. Cytosolic cytochrome c subsequently associates with apoptotic protease-activating factor (APAF-1) to form the apoptosome, which cleaves and activates proapoptotic caspases [74]. Apoptosis is modulated in part by antiapoptotic members of the BCL-2 protein family, which inhibit BAX and BAK activity and promote cell survival [75, 76]. Conversely, BH3-only proteins, such as BAD and BIM, stimulate cell death by activating BAX and BAK [77–79].

Evading apoptosis is an important hallmark of cancer, and the success of therapy depends, in large part, on its ability to induce cell death [80]. Many cancers, including breast, pancreatic, and endometrial cancer, evade apoptosis through upregulation of antiapoptotic proteins, including those in the BCL-2 protein family [81–84]. BCL-1, myeloid-cell leukemia (MCL-1), and B-cell lymphoma extralarge (BCL-XL) are antiapoptotic BCL-2 proteins frequently overexpressed in human cancer and protect cells against apoptosis under normal conditions in breast, prostate, and cholangiocarcinoma cancer cells [85–87]. In BRAF^V600E^-mutated cancers, MCL-1 has been shown to be aberrantly upregulated [58, 59]. Furthermore, constitutively active MAPK signalling phosphorylates and inactivates BAD and BIM, two proapoptotic BH3-only proteins (Figure 2(a)) [88, 89].

In recent years, co-opting proapoptotic pathways had been of interest in several cancer types, including melanoma and thyroid carcinoma [60, 90]. BH3 mimetics, single-agent small molecule inhibitors that have proapoptotic effects similar to BH3-only proteins, have shown efficacy in melanoma clinical trials for reducing tumour size [91]. Combining drugs that target proapoptotic BCL-2 family proteins with BRAF inhibitors in BRAF^V600E^-mutated cancers sensitizes melanoma cells to apoptosis but has not been shown to reverse resistance that is acquired over time [60]. However, Jeong et al. showed BRAFi therapy increased BCL-XL and BCL-2 expression in BRAF^V600E^-mutated human thyroid cancer cells [90]. Treating the same cells with BH3 mimetic, navitoclax, promoted apoptosis and growth inhibition compared with vemurafenib or navitoclax alone [90].

### 2.2. STAT3 is an Alternative Mechanism to Activate AntiaApoptotic Pathways

STAT proteins constitute a diverse array of transcription factors and are activated by a variety of extracellular signals, including epidermal growth factor (EGF), fibroblast growth factor (FGF), platelet-derived growth factor (PDGF), granulocyte-colony stimulating factor (G-CSF), interleukin-6 (IL-6), and insulin-like growth factor (IGF) [92–97]. STAT3 is activated via tyrosine phosphorylation and subsequently translocates to the nucleus where it activates transcription of genes involved in cell cycle progression including CCND1, c-MYC, and FOXM1 [98–100]. Interestingly, STAT3 also activates antiapoptotic BCL-2 family proteins, highlighting its dual involvement in prosurvival and apoptotic pathways [101].

In addition to BCL-2 family proteins, STAT3 represents an attractive target of antiapoptotic therapies. BRAF^V600E^ mutations are highly abundant in advanced-stage melanoma and colorectal cancer (CRC) [102, 103]. In tumours with acquired resistance to BRAFi therapy, STAT3 activation is associated with a poor prognosis [104, 105]. Alcolea recently developed and tested an organoseleneium compound, which reduced melanoma cell viability, suppressed proliferation, induced apoptosis, inhibited STAT3, and induced cell cycle inhibitor p21 [106]. This compound did not decrease phosphorylation of ERK1/2 or other relevant kinases, suggesting that it suppresses cell death through an alternative mechanism [106]. Dysregulation of apoptotic pathways may serve to increase the metastatic potential of BRAF^V600E^-mutated cancer cells [107, 108]. These data suggest that combining STAT3 inhibitors with BRAFi may be an effective way to overcome resistance in cancers that have failed BRAFi monotherapy.

## 3. Dual Roles of Autophagy in Cancer as Potential Therapeutic Targets

Autophagy is a process by which cells recycle the cytoplasmic material for energy use or biosynthesis of macromolecules. Importantly, autophagy plays dual roles in either promoting or preventing cell death. In healthy cells, autophagic processes are upregulated in response to stress, allowing for cell survival [109]. Accordingly, cells lacking autophagic capabilities cannot adapt to stressed environments and have a lower apoptotic threshold [110]. Despite its critical function in cell survival, autophagy also plays a role in cell death. In some cases, cells may overstimulate autophagy to catabolize indispensible cellular components, leading to “autophagic cell death” (also known as “Type II apoptosis”) [111]. High numbers of autophagosomes are often seen in dying cells, and there is evidence that autophagy alone is sufficient for cell death under certain conditions [112]. Pathway crosstalk between autophagy and apoptosis is explained in part by Beclin-1, a BH3-only protein that is critical in autophagy and interacts with antiapoptotic proteins BCL-2 and BCL-XL [113–115]. Proapoptotic caspase-mediated cleavage of Beclin-1 inhibits its autophagic effects and stimulates apoptosis through mitochondrial release of cytochrome c [115].

In relation to cancer, autophagy is complex and multifaceted. Similar to healthy tissue, autophagy plays dual roles in either promoting or inhibiting cancer cell death. Some evidence suggests that inhibition of autophagic processes can contribute to tumourigenesis [116, 117]. For example, the mammalian target of rapamycin (mTOR) negatively regulates autophagy, and consequently, inhibition of mTOR leads to cell death [118]. Similarly, stimulation of autophagy through other mechanisms, including intermittent fasting and AMPK activation, have been shown to inhibit tumour growth and selectively promote cancer cell death [119, 120]. However, in cancer, autophagic processes may be modulated to promote survival in conditions that would otherwise trigger programmed cell death [121]. During nutrient deprivation or metabolic stress, cells stimulate autophagy to survive, adapt to their environment, and evade cell death, which may result in oncogenesis by preventing senescence [122, 123]. Notably, cancer cells may co-opt normal autophagic processes to survive stressors in their microenvironment, suppress p53 function, and increase mitochondrial respiration [124–126]. In starved conditions, K-RAS-mutated cells become addicted to autophagy, allowing them to retain functional mitochondria [126]. RAS-mutated cells with abnormal autophagosomes accumulate defective mitochondria and have decreased oxygen consumption [126]. Interestingly, autophagy, mitophagy, and mitochondrial metabolism are attenuated during tumour formation, but can increase to maintain energy homeostasis and promote tumour survival in a stressed environment [126]. Taken together, autophagy serves dual functions; it either promotes apoptosis or cell survival. These functions are modulated based on the stage of tumour development, indicating that autophagy-targeted treatments should be tailored to the cancer phenotype.

In BRAF^V600E^-mutated cancer cells, autophagy may be upregulated as a protective mechanism in response to cellular stressors [61, 127, 128]. Recently, there have been preclinical studies using autophagy inhibitors in combination with BRAFi for BRAF^V600E^-mutated cancer cells [62, 129]. Kinsey et al. showed that inhibiting the RAF ⟶ MEK ⟶ ERK signalling cascade in K-RAS-driven cancers stimulated autophagy through activation of AMPK, a key energy sensor and metabolic regulator [62, 130]. In low glucose conditions, AMPK phosphorylates ULK1 at two serine residues leading to autophagosome formation and initiation of autophagy [130]. Conversely, under conditions of high nutrient availability, active mTOR phosphorylates ULK1 at a different serine residue, preventing its interaction with AMPK [130]. In preclinical models, the MEK1/2 inhibitor, trametinib, in combination with an autophagy inhibitor, chloroquine, demonstrated synergy in pancreatic ductal adenocarcinoma, colorectal carcinoma, and melanoma patient-derived xenograft (PDX) models with RAS and BRAF^V600E^ mutations [62].

Autophagy serves as an adaptive drug resistance mechanism in BRAF^V600E^-mutated cancers [61]. Ojha et al. showed that in melanoma, BRAFi therapy induces an ER stress response that stimulates autophagy and promotes cell survival. This stress response is mediated by protein-protein interactions between MAPK components (including BRAF^V600E^), chaperone protein GRP78, scaffolding protein KSR2, ER translocase SEC61, and early endosomes (Figure 2(b)) [129]. Activation of the ER stress response results in components of the MAPK pathway translocating to the ER via association with GRP78, KSR2, and SEC61 [129]. This translocation is required for ERK reactivation and subsequent stimulation of cytoprotective autophagy via ATF4 phosphorylation [129]. Accordingly, expression of mutant ATF4 has been shown to improve cellular sensitivity to MAPK inhibition *in vivo* [129]. Ryabaya et al. have shown that GRP78 blockade inhibits the ER stress-mediated autophagy and promotes apoptosis through caspase 7 to sensitize melanoma cells to temozolomide [131]. KP1339/IT-139, a GRP78 inhibitor, demonstrated significant anticancer activity in a Phase I clinical trial and is an attractive potential treatment for BRAFi-resistant tumours [132]. Taken together, these data provide rationale for clinical studies combining antiautophagic agents with BRAF and MEK inhibitors.

## 4. AKT, Copper Complexes, and Arachidonic Acid Metabolism Are Inflammatory Targets in BRAF^V600E^-Mutated Cancer

Lipid signalling in the phosphoinositide 3-kinase (PI3K)-AKT pathway plays a critical role in differentiation, cytoskeletal rearrangement, vesicle trafficking, growth, and mounting inflammatory responses [133, 134]. Membrane inositol phospholipids (PI) are modified by different classes of PI3K. PI3K has different isoforms, Class I, Class II, and Class III, which selectively phosphorylate membrane PIs [135, 136]. Importantly, Class I PI3Ks convert phosphatidylinositol-4,5-bisphosphate (PI(4,5)P2 or “PIP_2_”) to phosphatidylinositol-3,4,5-triphosphate (P(3,4,5)P3 or “PIP_3_”) [137, 138]. The PIP_3_ second messenger has many functions but classically activates the downstream kinase, AKT, which negatively regulates TSC1 and TSC2 heterodimers to modulate mTORC1 activity (Figure 2(b)) [139].

Inflammation and cancer are related bidirectionally, with high levels of chronic inflammation conferring an increased risk for tumour development [140, 141]. Conversely, cancer has been shown to alter surrounding tissue to suppress cancer-killing immune cells and promote chronic, proresolving inflammation [142, 143]. Tumour-infiltrating lymphocytes and macrophages constitute a significant population of cells in the microenvironment and modulate response to treatment [144, 145]. Cytokines associated with chronic inflammation, including TNF-*α*, TGF-*β*, and IL-6, remodel the microenvironment and promote tumourigenesis in part through generation of reactive oxygen species (ROS) [65, 146, 147]. The PI3K-AKT-mTOR signalling axis remodels surrounding tumour-stromal cells, playing an important role in tumour-infiltrating lymphocyte activity and oncogenesis [119]. PI3K signalling is often upregulated in BRAF^V600E^-mutated cells that are resistant to BRAFi therapy [148–151]. Isoforms of PI3K play important roles in lymphocyte chemotaxis and NK cell extravasation into tumour-stromal tissue [152, 153]. In addition, PI3K upregulates mTOR signalling, further driving oncogenesis (Figure 2(c)) [154]. Preclinical studies have successfully targeted PI3K signalling in combination with BRAF/MEK inhibitors in BRAF^V600E^-mutated colorectal cancer and melanoma cells [155–157]. BRAF^V600E^ melanoma cells treated to resistance with monotherapy BRAFi are sensitive to IGF-1R/PI3K and MEK inhibitors [155]. While single-agent vemurafenib is effective in less than 10% of BRAF^V600E^-mutated CRCs, the addition of PI3K inhibitors shows synergistic growth inhibition in drug-resistant CRC cells [156]. Jiang et al. showed BRAFi resistance increases tumour PD-L1 expression, which can be overcome by combined MEK and PI3K inhibition [157]. PI3K inhibitors in combination with vemurafenib have shown promising results in early clinical trials [158, 159]. In summary, PI3K inhibitors in combination with other treatments offer potential therapeutic benefit for BRAF^V600E^-mutated cancers resistant to monotherapy BRAFi.

In addition to classic pathway inhibition, other anti-inflammatory drugs have been tested preclinically with promising results. Copper is a tightly regulated cofactor for a wide variety of enzymes and is an essential micronutrient [160]. Copper dysregulation is seen in a variety of chronic diseases including Wilson's disease and Alzheimer's disease [161, 162]. Inflammatory ROS is seen with high serum copper [160, 163–166]. Elevated serum copper levels are correlated with poor survival in CRC and drug resistance in other tumour types [167]. In BRAF^V600E^-mutated lung cancer and melanoma cells, copper has been shown to enhance MEK1 phosphorylation of ERK1/2 through formation of a MEK1-copper complex [168]. Therapeutics targeting copper-driven ROS sequester block the uptake or chelate copper to mitigate ROS production [160, 167, 168]. Metallothionine and glutathione can protect against copper-driven ROS by sequestering it in the cytosol [160]. Copper uptake into the cell can be inhibited by targeting the CTR1 receptor and disrupting the MEK1-copper binding site to decrease ERK1/2 phosphorylation and downstream signalling [168]. Copper chelation therapy with tetrathiomolybdate is used in Wilson's disease and is being studied in cancers due to its antiangiogenic and anti-inflammatory properties [167]. In BRAFi resistant CRC cells, copper chelation therapy has been shown to decrease proliferation, survival, migration, and clonogenic potential [167]. These data highlight alternative approaches to overcoming resistance and developing drugs that address multiple cellular adaptations that contribute to oncogenesis (i.e., metabolism, generation of ROS, and oncogenic transformation).

One of the most widely used classes of drugs, nonsteroidal anti-inflammatory drugs (NSAIDs), has the potential to decrease the proliferative capacity of cancer [169, 170]. NSAIDs target cyclooxygenase (COX) enzymes, which play important roles in fever, pain, and inflammation. Membrane phospholipids are converted into arachidonic acid (AA) by either membrane phospholipase A2 (mPLA2) or soluble phospholipase A2 (sPLA2) [171–173]. AA is converted into lipid signalling molecules including prostaglandins, thromboxanes, and prostacyclins by COX1 and COX-2 isoenzymes [170]. In healthy tissues, COX1 is expressed and constitutively active, functioning as a “housekeeping” protein. Under ordinary conditions, COX1 produces thromboxane, prostacyclin, and prostaglandin E2, which serve normal functions in platelet aggregation and gastrointestinal cytoprotection. By contrast, COX-2 expression is induced during inflammation. COX-2 plays a critical role in mounting immune responses by producing prostaglandin E2, leading to fever and increased blood vessel permeability [170]. Classic inhibitors of COX1/2, including ibuprofen and naproxen, and more recent selective COX-2 inhibitors, including celecoxib, have been employed in clinical practice [174]. Targeting BRAF and the COX-2 orthogonal pathway could benefit patients with resistance to BRAFi drugs. Escuin-Ordinas et al. describe how celecoxib prevents the development of BRAFi-induced secondary cutaneous squamous cell carcinomas (cuSCCs) that result from paradoxical BRAF activation. In cuSCC cells, vemurafenib alone increased phosphorylation of ERK, a response that was decreased significantly by celecoxib. Furthermore, trametinib, a MEK inhibitor, reduced cuSCC development less efficiently than celecoxib. The authors suggest that reduced prostaglandin synthesis resulting from inhibition of COX-2 decreases the development of secondary cuSCCs [175]. The results of this study may inform us that targeting an orthogonal metabolic pathway could improve therapeutic efficacy of BRAFi therapy in BRAF^V600E^-mutated tumours. These data provide rationale for future investigations into inhibiting inflammatory processes in BRAF^V600E^-mutated cancers to overcome resistance to BRAFi therapy.

## 5. Energy Homeostasis in Resistant BRAF^V600E^-Mutated Cancer as a New Avenue for Therapy

Metabolic efficiency is frequently optimized to produce maximum ATP per molecule of glucose. To achieve maximum efficiency, healthy cells will metabolize glucose through oxidative phosphorylation in the mitochondria in the presence of oxygen. The flux through these metabolic pathways will be tightly regulated to meet the metabolic demands of any cell, modulate reactive oxygen formation, and keep metabolites at homeostatic abundancies through anaplerotic and cataplerotic reactions [176].

In 1931, Otto Warburg was awarded the Nobel Prize in Medicine and Physiology for his proposal that cancer cells metabolize glucose in the presence of oxygen, a process known as aerobic glycolysis [177–179]. This observation was named the Warburg Effect and has remained the classic paradigm of cancer metabolism [177, 180]. However, recent evidence suggests certain cancer types can uptake and oxidize lipids [181, 182]. Lipid catabolism is regulated by AMPK, a nutrient sensor of normal and cancer cells. AMPK phosphorylates and inactivates acetyl-CoA carboxylase, which impedes fatty acid synthesis and promotes lipid utilization by beta-oxidation in the mitochondria (Figure 2(d)) [183]. In fact, a recent study by Aloia has shown that upregulation of fat oxidation plays a role in the adaptive response to MAPK inhibition [184]. Particularly, the pharmacological blockade of CPT1A (the rate-limiting step for fat oxidation) reactivated glycolysis in MAPKi-treated melanoma cells. This compensatory increase in glycolytic flux in response to CPT1A inhibition has already been described and highlights the flexibility of cancer cell metabolism to promote resistance [185]. Thus, the concomitant inhibition of CPT1A, glycolysis, and MAPK synergistically inhibited tumour cell growth *in vitro* and in BRAF^V600E^-mutated melanoma mouse models [184].

Interestingly, AMPK phosphorylates BRAF at serine 729 [186]. Shen et al. showed that this phosphorylation leads to decreased MAPK signalling by preventing BRAF association with CRAF and KSR1 in BRAF wild type cells [186]. Consequently, AMPK presents an attractive therapeutic target for BRAFi-resistant tumours. However, the relationship between BRAF and AMPK is less clear in the context of BRAF^V600E^ mutations. Ritt et al. demonstrated that mutating serine 729 in BRAF^V600E^ cells did not affect MEK activation or transformation potential [187]. By contrast, mutating serine 729 in cells with intermediate BRAF kinase activity led to increased MEK activation [187]. While more investigation into the mechanism of AMPK regulation of BRAF^V600E^-mutated cells is necessary, AMPK inhibitors have shown efficacy in combination with BRAFi therapy [188]. Melanoma and CRC studies indicate that cancer cells increase their tolerance to MAPK pathway inhibition by activating AMPK-mediated autophagy [63, 189]. Consistent with these findings, a preclinical study by Yuan et al. demonstrated reduced drug resistance in melanoma cells treated with AMPK and BRAF inhibitor therapy compared with single-agent vemurafenib [188]. In contrast, a recent study found that BRAF inhibitors in combination with biguanide and phenformin (AMPK activators and complex I inhibitors) induced tumour regression [64]. Taken together, these studies merit further investigation into the relationship between BRAF and AMPK.

Metabolic reprogramming events may play a crucial role in tumourigenesis, drug resistance, and metastases [190]. A study on slow-cycling, chemotherapy-resistant BRAF^V600E^-mutated melanoma showed oxidative phosphorylation enzymes were upregulated, and consequently, their inhibition resulted in cell death [191]. Several compounds targeting mitochondrial respiration have been studied preclinically with promising results [192–194]. Molecular profiling shows enzymes involved in oxidative phosphorylation are enriched in melanoma brain metastases [193]. In a metastatic melanoma PDX study, *β*-sitosterol, an electron transport chain complex I inhibitor, effectively reduced oxidative phosphorylation and prevented the development of brain metastases [192]. Moreover, *β*-sitosterol increased the generation of ROS and consequently induced apoptosis [192]. Treatment with an oxidative phosphorylation inhibitor improved survival and decreased the development of brain metastases in BRAF/MEK inhibitor-resistant mice [193]. Uncoupling agents, which dissipate the proton gradient in the mitochondrial intermembrane space, have also inhibited tumour growth in vemurafenib-resistant melanoma PDX models [194]. Mechanistic studies indicate that uncouplers modulate mTOR and AMPK and induce apoptosis without perturbing MAPK signalling [194]. These data indicate that the metabolic profile of drug-resistant cancer cells clearly differs from drug sensitive subpopulations. Metabolic differences in lipid metabolism can be modulated therapeutically to overcome BRAFi resistance. The current paradigm and our understanding of the Warburg effect in cancer may be overlooking metabolic rewiring, such as lipid metabolism, and other adaptations in cancer.

## 6. Conclusions

Preclinical data on agents that work synergistically with MAPK therapy offer promising avenues for future clinical studies. Apoptosis, autophagy, and metabolism are particularly interesting areas of research and present opportunities for targeting nongenomic cancer transformations. Understanding the interconnectivity and regulation of these cellular processes will provide insight into the efficacy of targeted therapeutics in the context of tumour development, invasion, progression, and metastasis. So far, preclinical data show BRAF/MEK inhibitors in combination with alternative therapies are novel and efficacious approaches to overcoming BRAFi resistance. There have been advances in overcoming acquired resistance to BRAFi across cancers. However, it is important to acknowledge there are some leading fields, including quickly advancing therapies in melanoma, which could be beneficial for other tumour types and translated across cancers. Future research must elucidate mechanisms in which BRAFi-resistant cancers alter protein-protein interactions and their metabolism in order to develop rational targets for BRAF^V600E^-mutated cancers. Understanding the unique metabolic and autophagic profiles of BRAF^V600E^-mutated tumours across cancer types and disease stages will help to advance the development of therapeutics with lower toxicity than conventional treatments.

## Figures and Tables

**Table 1:** Summary pharmacological interventions for BRAFV600E mutated cancers^✝^.

Compound	Target	Pathway	Cancer type^*∗*^
Vemurafenib	BRAF	MAPK	CRC/G/M/T
Dabrafenib	BRAF	MAPK	CRC/M
Encorabenib	BRAF	MAPK	CRC/M
Trametinib	MEK	MAPK	CRC/M/PDA
Binimetinib	MEK	MAPK	M
Navitoclax	BCL-2/BCL-XL/BCLW	BH3 mimetic	M/T
ABT-737	BCL-2	BH3 mimetic	M
A-1210477	MCL-1	BH3 mimetic	M
Compound 1^+^	SH2	STAT3	M
Hydroxychloroquine	Unknown	Autophagy	CRC/M/PDA
Lys05	Lysosome	Lysosomal autophagy	M
Chloroquine	Unknown	Autophagy	CRC/M/PDA
Temozolomide	DNA	DNA replication	M
KP1339/IT-139	GRP78	ER homeostasis	MT
GSK2606414	PERK	UPR^#^	M
A443654.3	AKT	PI3K/AKT/mTOR	CC
MK2206	AKT	PI3K/AKT/mTOR	CRC
LY294002	PI3K	P3K/AKT/mTOR	CRC
GDC0941	PI3K	P3K/AKT/mTOR	CRC
PPP^++^	IGF-1R	PI3K/AKT/mTOR	M
Iso-orientin	Unknown	PI3K/AKT/Mitochondria	HBC
TM^+++^	Copper	Angiogenesis/inflammation	CRC
Ibuprofen	COX1/2	NSAID^##^	MT
Naproxen	COX1/2	NSAID^##^	MT
Celecoxib	COX-2	NSAID^##^	SCC
Etomoxir	CPT1A	Lipid oxidation	M/P
Phenformin	AMPK	Metabolic regulator	M
*β*-sitosterol	Complex I	ETC^###^	M
SR4	Proton uncoupler	Mitochondria	M
Niclosamide	Proton uncoupler	Mitochondria	M

^*∗*^CC = cholangiocarcinoma; CRC = colorectal carcinoma; G = glioma; HBC = hepatoblastoma cancer; M = melanoma; MT = multiple tumours; *P* = prostate; PDA = pancreatic ductal adenocarcinoma; PST = panel of solid tumours; SCC = squamous cell carcinoma; T = thyroid. ^+^Compound 1 = quinoxaline-2,3-diylbis (methylene) dicarbamimidoselenoate dihydrobromide. ^++^PPP = cyclolignan propodophyllin. ^++^TM = tetrathiomolybdate. ^#^UPR = unfolded protein response. ^##^NSAID = nonsteroidal anti-inflammatory. ^###^ETC = electron transport chain. ^✝^This is not a comprehensive list of compounds that target alternative mechanisms of BRAFi resistance and covers therapies that are referenced in this review.
